# Inter-rater and intra-rater agreement of [^99m^Tc]-labelled NM-01, a single-domain programmed death-ligand 1 (PD-L1) antibody, using quantitative SPECT/CT in non-small cell lung cancer

**DOI:** 10.1186/s13550-023-01002-4

**Published:** 2023-05-31

**Authors:** Daniel Johnathan Hughes, Gitasha Chand, Jessica Johnson, Damion Bailey, Kathryn Adamson, Vicky Goh, Gary J. R. Cook

**Affiliations:** 1 Department of Cancer Imaging, School of Biomedical Engineering and Imaging Sciences, King’s College London, London, UK; 2 King’s College London and Guy’s and St. Thomas’ PET Centre, Lambeth Wing, St Thomas’ Hospital, Westminster Bridge Road, London, SE1 7EH UK; 3 Department of Nuclear Medicine, Guy’s and St Thomas’ NHS Foundation Trust, London, UK; 4 Department of Radiology, Guy’s and St. Thomas’ NHS Foundation Trust, London, UK

**Keywords:** Technetium, SPECT, Non-small cell lung cancer, Immunotherapy, PD-L1, Single-domain antibody (sdAb)

## Abstract

**Background:**

Immune checkpoint inhibitors, including those against programmed cell death protein-1 (PD-1) or its ligand (PD-L1), are routinely used to treat non-small cell lung cancer (NSCLC). PD-L1 is a validated prognostic and predictive immunohistochemical biomarker of anti-PD-1/PD-L1 therapy but displays temporospatial heterogeneity of expression. Non-invasive radiopharmaceutical techniques, including technetium-99m [^99m^Tc]-labelled anti-PD-L1 single-domain antibody (NM-01) SPECT/CT, have the potential to improve the predictive value of PD-L1 assessment. This study aims to determine the inter- and intra-rater agreement of the quantitative measurement of [^99m^Tc]NM-01 SPECT/CT in NSCLC.

**Methods:**

Participants (*n* = 14) with untreated advanced NSCLC underwent [^99m^Tc]NM-01 SPECT/CT at baseline (*n* = 3) or at baseline plus 9-week follow-up (*n* = 11). [^99m^Tc]NM-01 uptake (of primary lung, lymph node, thoracic and distant metastases, and healthy reference tissues) was measured using SUV_max_ and malignant lesion-to-blood pool ratios with Siemens xSPECT Broad Quantification software by three independent raters. Intraclass correlation coefficients (ICC) were calculated and Bland–Altman plot analysis performed to determine inter- and intra-rater agreement.

**Results:**

There was excellent inter-rater agreement of manual freehand SUV_max_ scores of primary lung tumour (T; *n* = 25; ICC 1.00; 95% CI 0.99–1.00), individual lymph node metastases (LN; *n* = 56; ICC 0.97; 95% CI 0.95–0.98), thoracic metastases (ThMet; *n* = 9; ICC 0.94; 95% CI 0.83–0.99) and distant metastases (DisMet; *n* = 21; ICC 0.91; 95% CI 0.83–0.96). The inter-rater ICCs of tumour-to-blood pool (T:BP), LN:BP, ThMet:BP and DisMet:BP measures of [^99m^Tc]NM-01 uptake also demonstrated good or excellent agreement. Manual freehand scoring of T, LN, ThMet, DisMet and their ratios using [^99m^Tc]NM-01 SPECT/CT following a 28-day interval was consistent for all raters with good or excellent intra-rater agreement demonstrated (ICCs range 0.86–1.00).

**Conclusion:**

Quantitative assessment of [^99m^Tc]NM-01 SPECT/CT in NSCLC, using SUV_max_ of malignant primary or metastatic lesions and their ratios with healthy reference tissues, demonstrated good or excellent inter- and intra-rater agreement in this study. Further validation with ongoing and future larger cohort studies is now warranted.

**Clinical trial registration:**

ClinicalTrials.gov identifier no. NCT04436406 (registered 18th June 2020; available at https://clinicaltrials.gov/ct2/show/NCT04436406) and NCT04992715 (registered 5th August 2021; available at https://clinicaltrials.gov/ct2/show/NCT04992715).

**Supplementary Information:**

The online version contains supplementary material available at 10.1186/s13550-023-01002-4.

## Background

Lung cancer is the second most commonly diagnosed cancer and a major cause of mortality globally with over 1.8 million deaths in 2020 alone [1]. Over the past decade, the treatment paradigm of advanced non-small cell lung cancer (NSCLC) has shifted with the introduction of therapies targeting immune checkpoint molecules, including programmed cell death protein 1 (PD-1) and its ligand (PD-L1). An important mechanism of immune escape involves the upregulation of co-inhibitory molecule PD-L1 by tumour cells, which on interaction with PD-1, expressed by effector T cells, lead to their dysfunction. PD-1/PD-L1 monoclonal antibodies are now widely used in the management of advanced NSCLC with significant improvements in median overall survival demonstrated in both first- and second-line treatment compared to standard cytotoxic chemotherapy [2–5]. Importantly, even in the advanced setting, durable responses can be seen in around 20% of patients [6].

Anti-PD-1/PD-L1 therapeutic response and survival in NSCLC are associated with PD-L1 tumour proportion score (TPS) measured using immunohistochemistry, a widely available and validated biomarker [2–7]. Those with metastatic NSCLC and a PD-L1 TPS ≥ 50% are likely to respond to single agent immunotherapy, such as anti-PD-1 pembrolizumab, whereas in those with < 1% (negative) or 1–49% (low) TPS, first-line treatment would usually include a combination approach with cytotoxic chemotherapy [2, 6, 8]. This ability to predict response allows many patients to avoid the treatment burden and toxicity associated with combination therapy. However, up to 10% of patients deemed ‘non-expressors’ by immunohistochemistry may respond to anti-PD-1/PD-L1 therapy and vice versa, resulting in a proportion of patients potentially being over- or under-treated [3]. Importantly, PD-L1 expression within and between tumours is heterogenous, as well as being dynamic, with changes over time particularly following exposure to anti-cancer therapies [9, 10]. Mapping PD-L1 expression within and across tumour sites over the course of an individual’s cancer journey with multiple and serial biopsies is impractical and exposes them to additional risk. Non-invasive imaging techniques present a potential solution in overcoming the limitations of PD-L1 expression measured by immunohistochemistry and provide opportunity to improve the predictive value of PD-L1 assessment.

The 14.3 kDa camelid single-domain PD-L1 antibody, NM-01, can be radiolabelled with technetium-99m ([^99m^Tc]), administered and subsequently detected by single-photon emission computed tomography (SPECT). A first-in-human study of [^99m^Tc]NM-01 demonstrated both safety and acceptable dosimetry in 16 participants with NSCLC [11]. SPECT/computed tomography (CT) scans were obtained 1 and 2 h following [^99m^Tc]NM-01 injection with 2-h primary tumour-to-blood pool ratio (T:BP) assessment correlating with PD-L1 immunohistochemistry. [^99m^Tc]NM-01 uptake greater than blood pool was also demonstrated in nodal and bone metastases with intertumoural heterogeneity in 30% of participants. As such, non-invasive PD-L1 assessment using this novel single-domain antibody could help oncologists better stratify patients to receive the most appropriate anti-cancer therapy at the right time in their disease course and facilitate the advancement of novel imaging biomarker driven clinical trials. We hypothesised that PD-L1 expression measured using [^99m^Tc]NM-01 with a quantitative SPECT/CT imaging approach is consistent and reproducible between individual raters and over time. Our aim was to determine the inter-rater and intra-rater agreement of [^99m^Tc]NM-01 uptake measurements in experienced and less experienced raters in nuclear medicine within a cohort of patients with NSCLC.

## Methods

Participants in the PD-L1 Expression in Cancer (PECan; NCT04436406) and PD-L1 Expression in Lung Cancer (PELICAN; NCT04992715) PD-L1 imaging studies scheduled to undergo [^99m^Tc]NM-01 SPECT/CT imaging were included in this single-centre prospective study conducted at Guy’s and St Thomas’ NHS Foundation Trust, London, UK. Participants aged 18 years and over with histologically confirmed, untreated advanced NSCLC scheduled for systemic anti-cancer therapy, tissue available for PD-L1 analysis and an Eastern Cooperative Oncology Group (ECOG) performance score of 1 or less were eligible. Exclusion criteria included pregnant or lactating females, severe infection, and prognosis of < 3 months. All participants provided written informed consent. Participants in the PECan study underwent [^99m^Tc]NM-01 SPECT/CT at baseline, prior to starting immune checkpoint inhibitor containing regimen, and at 9-week follow-up. Participants in the PELICAN study underwent [^99m^Tc]NM-01 SPECT/CT at baseline only and were eligible to participate independently of planned systemic therapy. Diagnostic samples of all recruited participants underwent PD-L1 immunohistochemical assessment as standard of care using the Ventana PD-L1 (SP263) assay.

### SPECT/CT protocol

SPECT/CT examinations were performed on a Siemens Symbia Intevo Bold SPECT/CT scanner calibrated for use of xSPECT Broad Quantification quantitative analysis software (Siemens Healthcare GmBH; Erlangen, Germany). Participants (*n* = 14) were administered an intravenous bolus of [^99m^Tc]NM-01, median 584 MBq (range 343–721 MBq) [^99m^Tc] corresponding to approximately 100 μg of NM-01. Participants were asked to drink 500 mL water post-injection and void their bladder prior to imaging. Whole-body planar imaging was performed with the patient supine at 2 h post-injection using a 256 × 1024 matrix at 10 cm/slice/min. Single field of view SPECT/CT imaging, focussing on primary tumour (thorax) and site(s) of suspected metastases was subsequently performed with the patient supine. Scans were acquired on a 256 × 256 matrix using low-energy high-resolution collimators, with a 15% energy window centred at 140 keV (the Tc-99m photopeak). A 15% energy window centred at 120 keV was also used for tomographic image acquisition for scatter correction. SPECT was performed over 180° with 128 projections (64 views), in step and shoot mode, 20 s per projection. A low-dose CT (130 kV, effective mAs determined using CARE Dose4D) was performed for anatomical correlation and attenuation correction. Images were reconstructed within an xSPECT Broad Quantification reconstruction workflow using OSEM iterative reconstruction (2 iterations, 10 subsets) with an additive update mechanism, at a matrix size of 128 × 128, with scatter correction. An xSPECT Broad Quantification analysis workflow was then utilised for the presentation of quantitative uptake data, gathered utilising inputted injected activity and patient weight [12].

### Image analysis

Images were reviewed by three independent raters blinded to patient details and each other’s assessments using Hermes GOLD™ (Hermes Medical Solutions; Stockholm, Sweden). The raters included 1 nuclear medicine physician, 1 nuclear medicine clinical fellow and 1 oncology clinical fellow PhD student with 30-, 5- and 3-year experience in nuclear medicine image analysis, respectively. Regions of interest, including primary tumour, metastatic lesions (including thoracic lymph nodes, thoracic metastases—pleural or lung, and distant metastases), and normal tissue references (lung, blood pool, bone marrow, liver and spleen), were identified on fused SPECT/CT. SUV_max_ was chosen as it represents the highest voxel within a given region of interest and is independent of the exact definition of the region, as long as the highest voxel is included in the region. Whilst SUV_mean_, the average SUV across all voxels in a region, is less sensitive to noise, it could be significantly affected by differences in the manual segmentation and thus subject to greater inter- and intra-rater variability [13]. It is also more likely to be affected by the partial volume effect, which is of particular importance when measuring small tumours and lymph nodes in this study of early NSCLC. Additionally, the previously reported study of [^99m^Tc]NM-01 SPECT/CT demonstrated maximum region of interest (ROI_max_) correlated with PD-L1 immunohistochemistry [11]. Although quantitative SPECT/CT permits the measurement of SUV_max_ for lesions, its methods are not fully validated and may be subject to less accuracy than PET/CT, such as that due to the partial volume effect associated with small regions of interest. As such, ratios of primary tumour and metastatic lesions to healthy reference tissues were also evaluated to allow for analysis on SPECT scanners that do not have the ability to calculate SUV_max_, as with the previously reported study of [^99m^Tc]NM-01 SPECT/CT.

Using a freehand manual technique, the maximum standardised uptake values (SUV_max_) were recorded from the SPECT images (*n* = 25; baseline only images in *n* = 3, baseline and 9-week follow-up images in *n* = 11). Freehand SUV_max_ was recorded for normal lung in the right upper lobe (or contralateral upper lobe if pathology present) for calculation of tumour-to-lung (T:L) ratio and for blood pool within the aortic arch for calculation of tumour-to-blood pool (T:BP) and metastatic lesion-to-blood pool ratios. Similarly, freehand SUV_max_ was measured for normal haematopoietic tissues, including bone marrow (thoracic vertebra at the level of the carina), spleen and liver.

**Fig. 1:**
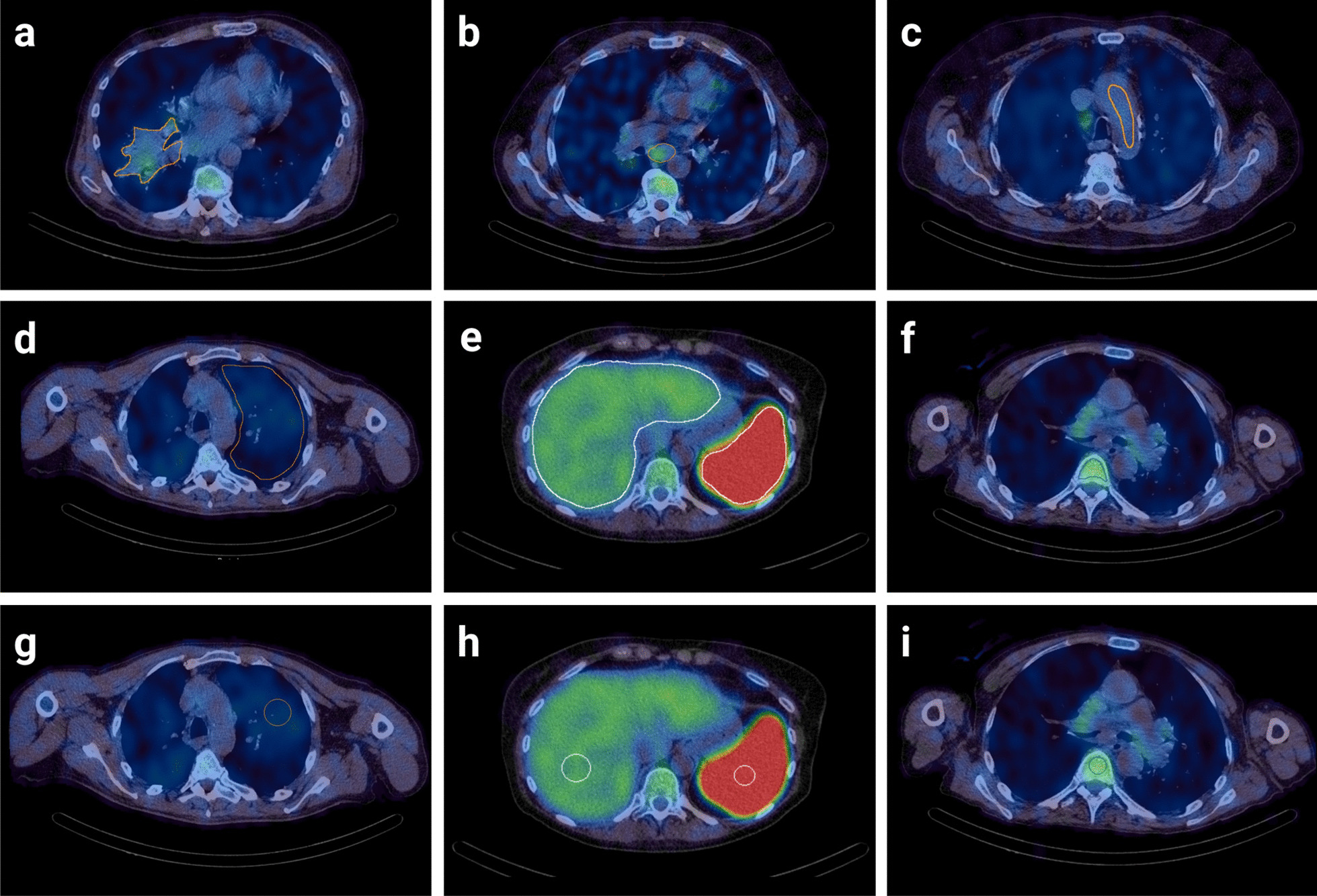
Image analysis using SUV_max_ scoring of [^99m^Tc]NM-01 SPECT/CT. **a** Freehand manual region of interest of a baseline (pre-treatment) primary right lower lobe tumour. **b** Freehand manual region of interest of a baseline station 7 (subcarinal) thoracic lymph node. **c** Blood pool SUV_max_ measured using a freehand manual technique. Healthy reference tissue SUV_max_ measurements determined by a freehand approach of **d** left upper lobe lung, **e** liver and spleen, and **f** bone marrow. Healthy reference tissue SUV_max_ measurements determined by volumetric rule-based approaches of **g** left upper lobe (3 cm sphere at level of aortic arch), **h** liver (3 cm sphere at level of the GOJ) and spleen (2 cm sphere), and **i** bone marrow (1.5 cm sphere at level of the carina)

To evaluate if rule-based approaches improved consistency of scoring normal tissue references, SUV_max_ was measured using a standardised 3-cm-diameter sphere for normal lung at the level of the aortic arch and carina, and the liver at the level of the gastroesophageal junction (GOJ) on axial views. SUV_max_ was measured using a standardised 2-cm-diameter sphere for the spleen and using a 1.5-cm-diameter sphere for the bone marrow in the thoracic vertebra at the level of the carina on axial views. Examples of image analysis are provided in Fig. 1. To determine intra-rater agreement, the 3 independent raters repeated their calculations for all measured regions blind to their initial measurements following a minimum 28-day period.

### Statistical analysis

The intraclass correlation coefficient (ICC) is a widely used reliability index representing both the degree of correlation and the agreement between measurements. ICC values range from 0 to 1, where less than 0.5 indicates poor agreement, 0.5 to < 0.75 moderate, 0.75 to < 0.9 good and values greater than 0.9, i.e. close to 1 represent excellent agreement [14]. A two-way random consistent model was used to determine the ICC and its 95% confidence intervals (CI) for inter-rater agreement of all 3 raters. A two-way mixed effects absolute agreement model was used to determine the ICC and its 95% CI for intra-rater agreement for each rater. Each level of agreement is more accurately defined by their 95% confidence intervals, considering the ICC is an estimated reliability index. Normality was assessed using the Shapiro–Wilk test, and non-normally distributed data were log transformed. One-sample *t* test was used to assess the difference of means (where two-sided *p* < 0.05 is significant). Bland–Altman plots and their 95% limits of agreement were used to determine the agreement between raters and their repeat measurements for T:BP, LN:BP, thoracic metastasis-to-blood pool ratio (ThMet:BP) and distant metastasis-to-blood pool ratio (DisMet:BP) scores. Linear regression of Bland–Altman plots was performed to determine the *β* coefficient of the mean difference and demonstrate any proportional bias (where *p* < 0.05 is significant). Statistical analysis was performed using IBM SPSS Statistics for Windows, version 28.0 (Armonk, NY: IBM Corp).

## Results

### Participant characteristics

Participants were recruited to the study between October 2020 and September 2022 (*n* = 14). The median age was 64 years (range 52–75 years); all were of white ethnicity. All had a histologically confirmed diagnosis of NSCLC (adenocarcinoma *n* = 10, squamous cell carcinoma *n* = 2, not otherwise specified *n* = 2) with 12 participants having metastatic disease at diagnostic staging. Detailed and summarised participant characteristics are provided in Table 1 and Additional file 1: Table S1.

**Table 1:** Summary of participant characteristics

Clinical characteristic	Total *n* = 14
*Age (years)*	
Median	64
Range	52–75
*Sex, n (%)*	
Female	6 (43)
Male	8 (57)
*Ethnicity, n (%)*	
White (British, Irish, other)	14 (100)
Black (African, British, Carribean)	0 (0)
Asian (Asian, British)	0 (0)
other	0 (0)
*Smoking status, n (%)*	
Never smoker	0 (0)
Ex-smoker	10 (72)
Smoker	4 (28)
Unknown	0 (0)
*ECOG PS, n (%)*	
0	3 (21)
1	11 (79)
*Histopathology, n (%)*	
NSCLC-adenocarcinoma	10 (72)
NSCLC-squamous cell carcinoma	2 (14)
NSCLC-NOS	2 (14)
*PD-L1 TPS, n (%)*	
< 1%	5 (36)
1–49%	1 (7)
≥ 50%	8 (57)
*Metastatic status, n (%)*	
M0 or Mx	2 (14)
M1a	5 (36)
M1b	4 (29)
M1c	3 (21)

Metastatic status is according to baseline diagnostic TNM staging according to 8th Edition of TNM in Lung Cancer. See Additional file 1: Table S1 for individual participant demographics including TNM staging, disease sites and administered radioactivity

*ECOG* Eastern Cooperative Oncology Group Performance score, *NOS* not otherwise specified, *NSCLC* non-small cell lung cancer, *PD-L1* programmed death-ligand 1, *TPS* tumour proportion score

### Inter-rater agreement

There was excellent agreement of manual freehand SUV_max_ scores between all three raters of the primary lung tumour (T; *n* = 25; ICC 1.00; 95% CI 0.99–1.00) and individual lymph node metastases (LN; *n* = 56; ICC 0.97; 95% CI 0.95–0.98) (Table 2). There was good to excellent inter-rater agreement for freehand SUV_max_ measurements of individual thoracic metastases (ThMet; *n* = 9; ICC 0.94; 95% CI 0.83–0.99) and distant metastases (DisMet; *n* = 21; ICC 0.91; 95% CI 0.83–0.96).

**Table 2:** Inter-rater agreement of [^99m^Tc]NM-01 measurements

	Rater A	Rater B	Rater C		
SPECT	SUV_max_ (mean ± SD)	SUV_max_ (mean ± SD)	SUV_max_ (mean ± SD)	ICC (95% CI)	ICC Level of agreement
*Malignant lesion(s) SUV*_*max*_					
Primary lung tumour (T)	3.58 ± 1.27	3.57 ± 1.28	3.61 ± 1.25	1.00 (0.99–1.00)	Excellent
Lymph node metastasis (LN)	3.23 ± 1.50	3.19 ± 1.53	3.33 ± 1.49	0.97 (0.95–0.98)	Excellent
Thoracic metastasis	2.72 ± 1.62	2.79 ± 1.24	2.64 ± 1.05	0.94 (0.83–0.99)	Good to excellent
Distant metastasis	4.30 ± 2.05	4.25 ± 2.00	4.49 ± 2.21	0.91 (0.83–0.96)	Good to excellent
*Reference tissues*					
Blood pool (BP)	1.43 ± 0.50	1.46 ± 0.48	1.39 ± 0.43	0.87 (0.76–0.94)	Good to excellent
Lung (freehand)	1.72 ± 0.58	1.69 ± 0.57	1.37 ± 0.49	0.93 (0.86–0.96)	Good to excellent
Lung (volume AA)	1.38 ± 0.53	1.38 ± 0.50	1.35 ± 0.59	0.88 (0.78–0.94)	Good to excellent
Lung (volume C)	1.39 ± 0.49	1.34 ± 0.55	1.42 ± 0.51	0.94 (0.88–0.97)	Good to excellent
Liver (freehand)	6.26 ± 1.34	6.56 ± 1.33	6.27 ± 1.24	0.93 (0.87–0.97)	Good to excellent
Liver (volume GOJ)	5.28 ± 1.03	5.25 ± 1.21	5.27 ± 1.01	0.90 (0.81–0.95)	Good to excellent
Spleen (freehand)	20.11 ± 4.18	20.47 ± 3.97	20.68 ± 3.71	0.90 (0.82–0.95)	Good to excellent
Spleen (volume)	18.89 ± 3.45	19.1 ± 3.62	19.18 ± 3.67	0.94 (0.88–0.97)	Good to excellent
Bone marrow (freehand)	3.35 ± 0.80	3.52 ± 0.82	3.44 ± 0.86	0.90 (0.82–0.95)	Good to excellent
Bone marrow (volume)	3.36 ± 0.79	3.50 ± 0.83	3.36 ± 0.86	0.93 (0.87–0.97)	Good to excellent

	SUV_max_ ratio (mean ± SD)	SUVmax ratio (mean ± SD)	SUV_max_ ratio (mean ± SD)	ICC (95% CI)	ICC Level of agreement
*Ratios*					
T:BP	2.60 ± 0.82	2.56 ± 0.87	2.68 ± 0.78	0.85 (0.73–0.93)	Moderate to excellent
T:L (freehand)	2.23 ± 0.92	2.27 ± 0.91	2.87 ± 1.16	0.91 (0.83–0.96)	Good to excellent
T:L (AA)	2.89 ± 1.24	2.83 ± 1.14	3.04 ± 1.42	0.88 (0.79–0.94)	Good to excellent
T:L (C)	2.80 ± 1.10	2.99 ± 1.28	2.83 ± 1.36	0.89 (0.80–0.95)	Good to excellent
LN:BP	2.57 ± 1.43	2.37 ± 1.28	2.52 ± 1.15	0.90 (0.85–0.94)	Good to excellent
Thoracic Met:BP	2.17 ± 1.11	2.27 ± 1.25	2.17 ± 0.90	0.94 (0.81–0.98)	Good to excellent
Distant Met:BP	2.80 ± 1.62	2.94 ± 1.75	3.13 ± 1.81	0.88 (0.77–0.94)	Good to excellent
Spleen:Liver	3.33 ± 0.99	3.21 ± 0.74	3.37 ± 0.69	0.78 (0.63–0.89)	Moderate to good
Bone marrow:Liver	0.55 ± 0.14	0.55 ± 0.13	0.56 ± 0.13	0.86 (0.75–0.93)	Good to excellent

Primary tumour, thoracic lymph node, thoracic or distant metastases and healthy tissue reference measurements (SUV_max_; mean ± SD) and their ratios of all three raters with intraclass correlation coefficient (ICC), their 95% confidence interval (CI) and descriptive ICC level of agreement

*AA* aortic arch, *BP* blood pool, *C* carina, *CI* confidence interval, *ICC* intraclass correlation coefficient, *GOJ* gastroesophageal junction, *L* lung, *LN* lymph node metastasis, *ROI* region of interest, *T* primary lung tumour

Freehand SUV_max_ measurements of blood pool (BP; *n* = 25; ICC 0.87; 95% CI 0.76–0.94) and lung (L; *n* = 25; ICC 0.93; 95% CI 0.86–0.96) normal reference tissues demonstrated good to excellent inter-rater agreement. The ICC using the volumetric rule-based approach measuring the SUV_max_ of normal lung at the level of the aortic arch (ICC 0.88; 95%CI 0.78–0.94) and the carina (ICC 0.94; 95% CI 0.88–0.97) also demonstrated good to excellent inter-rater agreement. Calculated T:L ratios demonstrated good to excellent inter-rater agreement with all methods (Table 2).

There was good to excellent inter-rater agreement of manual freehand SUV_max_ measurements of the normal haematopoietic reference tissues, including of the liver (*n* = 25; ICC 0.93; 95% CI 0.87–0.97), spleen (*n* = 25; ICC 0.90; 95% CI 0.82–0.95) and bone marrow (*n* = 25; ICC 0.90; 95% CI 0.82–0.95). Applying a rule-based volumetric approach to measure the SUV_max_ of the liver (ICC 0.90; 95% CI 0.81–0.95), spleen (ICC 0.94; 95% CI 0.88–0.97) and bone marrow (ICC 0.93; 95% CI 0.87–0.97) demonstrated good to excellent inter-rater agreement. Spleen-to-liver (SLR; ICC 0.78; 95% CI 0.63–0.89) and bone marrow-to-liver ratios (BLR; ICC 0.86; 95% CI 0.75–0.93) both demonstrated good inter-rater agreement.

**Fig. 2:**
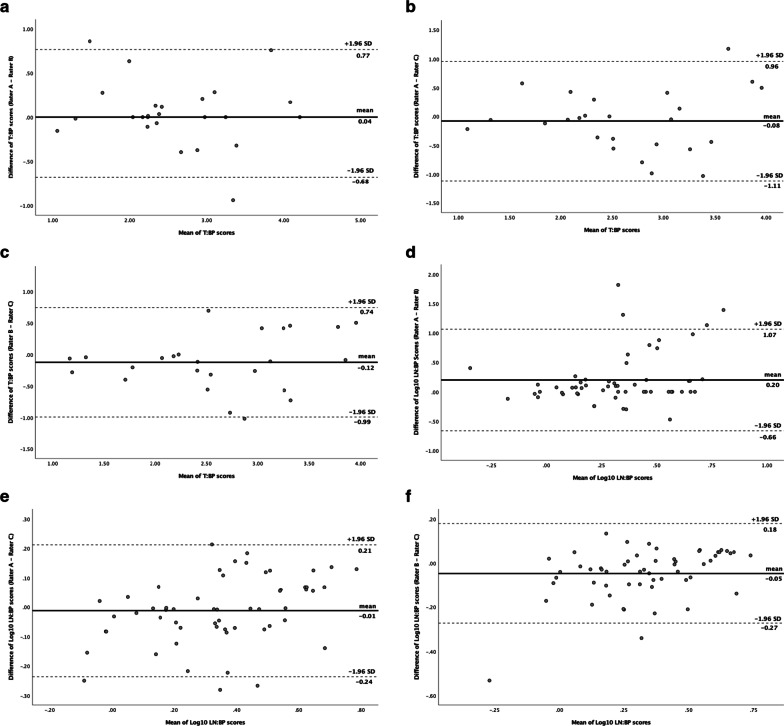
Inter-rater Bland–Altman level of agreement plots for T:BP (**a**–**c**) and log_10_ LN:BP (**d**–**f**) scores. Solid horizontal lines represent between-rater mean difference. Upper and lower 95% limits of agreement are represented by dashed lines. **a** T:BP scores of rater A versus B (t-test *p* = 0.55; *β* = -0.06, *p* = 0.51); **b** T:BP scores rater A versus C (*p* = 0.46; *β* = 0.05, *p* = 0.74); **c** T:BP scores rater B versus C (*p* = 0.18; *β* = 0.11, *p* = 0.34); **d** LN:BP scores rater A versus B (*p* < 0.05; *β* = 0.51, *p* < 0.05); **e** LN:BP scores rater A versus C (*p* = 0.44; *β* = 0.20, *p* < 0.05); **f** LN:BP scores rater B versus C (*p* < 0.05; *β* = 0.22, *p* < 0.05)

Malignant lesion-to-blood pool ratios used as a quantitative measure of [^99m^Tc]NM-01 uptake demonstrated good or excellent inter-rater agreement. T:BP (ICC 0.85; 95%CI 0.73–0.93), LN:BP (ICC 0.90; 95% CI 0.85–0.94) and DisMet:BP (ICC 0.88; 95% CI 0.77–0.94) all demonstrated good inter-rater agreement. ThMet:BP (ICC 0.94; 95% CI 0.81–0.98) demonstrated excellent inter-rater agreement. Bland–Altman plot analysis demonstrated inter-rater agreement with no proportional bias on linear regression for T:BP scores (Fig. 2) and DisMet:BP scores (Additional file 2: Fig. S1). However, one-sample t-test of LN:BP scores demonstrated statistical difference between the means of rater A versus B (*p* < 0.05) and rater B versus C (*p* < 0.05). The Bland–Altman analysis plots and associated *β* coefficients for LN:BP scores are included (Fig. 2). There was no statistical significance, and as such, acceptable agreement between the means of rater A versus C (*p* = 0.04) for LN:BP scores; however, there was proportional bias on linear regression (*β* = 0.20, *p* < 0.05). Inter-rater agreement was demonstrated for ThMet:BP scores; however, with proportional bias for rater B versus C (*β* = 0.34, *p* < 0.05; Additional file 2: Fig. S1).

### Intra-rater agreement

Freehand SUV_max_ scoring using [^99m^Tc]NM-01 SPECT/CT following a 28-day interval of all malignant lesions including primary lung tumour, lymph node, thoracic and distant metastases was consistent for all 3 raters (Table 3). The intra-rater ICC for primary lung tumour (T; *n* = 25) SUV_max_ measurements for rater A (ICC 1.00; 95% CI 0.99–1.00), rater B (ICC 1.00; 95% CI 1.00–1.00), and rater C (ICC 1.00; 95% CI 1.00–1.00) demonstrated excellent agreement. SUV_max_ measurements for lymph node (*n* = 56; ICCs 0.99–1.00), thoracic (*n* = 9; ICCs 0.97–0.98) and distant (*n* = 21; ICCs 0.94–0.99) metastases demonstrated excellent intra-rater agreement for all raters (see Table 3 for individual intra-rater ICCs and their 95% CI).

**Table 3:** Intra-rater agreement of [^99m^Tc]NM-01 measurements

SPECT	Rater A			Rater B			Rater C		
	1 SUV_max_ (mean ± SD)	2 SUV_max_ (mean ± SD)	ICC (95% CI)	1 SUV_max_ (mean ± SD)	2 SUV_max_ (mean ± SD)	ICC (95% CI)	1 SUV_max_ (mean ± SD)	2 SUV_max_ (mean ± SD)	ICC (95% CI)
*Malignant lesion(s)*									
Primary lung tumour (T)	3.58 ± 1.27	3.62 ± 1.24	1.00 (0.99–1.00	3.57 ± 1.28	3.56 ± 1.30	1.00 (1.00–1.00)	3.62 ± 1.25	3.61 ± 1.25	1.00 (1.00–1.00)
Lymph node metastasis (LN)	3.23 ± 1.50	3.23 ± 1.51	0.99 (0.99–1.00)	3.19 ± 1.53	3.22 ± 1.53	0.99 (0.99–1.00)	3.36 ± 1.47	3.33 ± 1.49	1.00 (0.99–1.00)
Thoracic metastasis	2.72 ± 1.16	2.80 ± 1.20	0.97 (0.89–0.99)	2.79 ± 1.24	2.65 ± 1.09	0.97 (0.88–0.99)	2.70 ± 1.19	2.64 ± 1.05	0.98 (0.92–1.00)
Distant metastasis	4.30 ± 2.05	4.25 ± 2.00	0.97 (0.93–0.99)	4.25 ± 2.00	4.02 ± 2.19	0.94 (0.86–0.98)	4.42 ± 2.09	4.50 ± 2.21	0.99 (0.98–1.00)
*Reference tissues*									
Blood pool (BP)	1.43 ± 0.50	1.47 ± 0.42	0.90 (0.80–0.96)	1.46 ± 0.48	1.42 ± 0.46	0.98 (0.95–0.99)	1.35 ± 0.37	1.39 ± 0.43	0.97 (0.93–0.99)
Lung (freehand)	1.72 ± 0.58	1.80 ± 0.62	0.90 (0.79–0.96)	1.69 ± 0.57	1.61 ± 0.52	0.86 (0.70–0.93)	1.38 ± 0.50	1.37 ± 0.49	1.00 (0.99–1.00)
Lung (AA)	1.38 ± 0.53	1.34 ± 0.48	0.96 (0.90–0.98)	1.38 ± 0.50	1.37 ± 0.52	0.96 (0.91–0.98)	1.36 ± 0.58	1.35 ± 0.59	0.99 (0.97–0.99)
Lung (C)	1.39 ± 0.49	1.38 ± 0.49	0.98 (0.95–0.99	1.34 ± 0.55	1.33 ± 0.45	0.94 (0.86–0.97)	1.46 ± 0.53	1.42 ± 0.51	0.98 (0.96–0.99)
Liver (freehand)	6.26 ± 1.34	6.29 ± 1.30	0.94 (0.86–0.97)	6.56 ± 1.33	6.66 ± 1.29	0.99 (0.96–0.99)	6.28 ± 1.23	6.27 ± 1.24	0.99 (0.99–1.00)
Liver (GOJ)	5.28 ± 1.03	5.27 ± 1.10	0.90 (0.79–0.96)	5.25 ± 1.21	5.29 ± 1.27	0.98 (0.96–0.99)	5.30 ± 1.06	5.27 ± 1.01	0.96 (0.92–0.98)
Spleen (freehand)	20.11 ± 4.18	21.02 ± 4.25	0.91 (0.76–0.97)	20.47 ± 3.97	20.57 ± 3.91	0.99 (0.98–1.00)	21.11 ± 3.84	20.68 ± 3.71	0.97 (0.91–0.99)
Spleen (volume)	18.89 ± 3.45	18.97 ± 3.53	0.95 (0.89–0.98)	19.10 ± 3.62	19.10 ± 3.51	0.96 (0.90–0.98)	19.38 ± 3.75	19.18 ± 3.67	0.97 (0.93–0.99)
Bone marrow (freehand)	3.35 ± 0.80	3.39 ± 0.78	0.91 (0.80–0.96)	3.52 ± 0.82	3.51 ± 0.83	0.99 (0.97–0.99)	3.55 ± 0.75	3.44 ± 0.86	0.90 (0.79–0.96)
Bone marrow (volume)	3.36 ± 0.79	3.38 ± 0.78	0.91 (0.80–0.96)	3.50 ± 0.83	3.52 ± 0.84	0.98 (0.96–0.99)	3.50 ± 0.77	3.36 ± 0.86	0.91 (0.79–0.96)

	SUV_max_ ratio (mean ± SD)	SUV_max_ ratio (mean ± SD)	ICC (95% CI)	SUV_max_ ratio (mean ± SD)	SUV_max_ ratio (mean ± SD)	ICC (95% CI)	SUV_max_ ratio (mean ± SD)	SUV_max_ ratio (mean ± SD)	ICC (95% CI)
*Ratios*									
T:BP	2.60 ± 0.82	2.52 ± 0.74	0.86 (0.71–0.94)	2.56 ± 0.87	2.61 ± 0.89	0.97 (0.92–0.98)	2.73 ± 0.77	2.68 ± 0.78	0.99 (0.97–0.99)
T:L (freehand)	2.35 ± 0.92	2.12 ± 0.89	0.87 (0.74–0.94)	2.27 ± 0.91	2.39 ± 1.04	0.92 (0.82–0.96)	2.86 ± 1.17	2.87 ± 1.16	1.00 (0.99–1.00)
T:L (AA)	2.89 ± 1.24	3.01 ± 1.28	0.97 (0.94–0.99)	2.83 ± 1.14	2.89 ± 1.22	0.96 (0.92–0.98)	3.03 ± 1.39	3.04 ± 1.42	0.99 (0.97–0.99)
T:L (C)	2.80 ± 1.10	2.85 ± 1.12	0.96 (0.92–0.98)	2.99 ± 1.28	2.90 ± 1.17	0.95 (0.89–0.98)	2.73 ± 1.20	2.83 ± 1.36	0.97 (0.93–0.99)
LN:BP	2.57 ± 1.43	2.35 ± 1.27	0.94 (0.87–0.97)	2.37 ± 1.28	2.50 ± 1.34	0.98 (0.95–0.99)	2.57 ± 1.10	2.52 ± 1.15	0.98 (0.97–0.99)
Thoracic met:BP	2.17 ± 1.11	2.06 ± 0.90	0.95 (0.82–0.99)	2.27 ± 1.25	2.04 ± 1.06	0.95 (0.72–0.99)	2.24 ± 1.01	2.17 ± 0.90	0.99 (0.94–1.00)
Distant met:BP	2.80 ± 1.62	2.84 ± 1.41	0.89 (0.75–0.96)	2.94 ± 1.75	2.86 ± 2.09	0.96 (0.91–0.98)	3.12 ± 1.65	3.13 ± 1.81	0.98 (0.96–0.99)
Spleen:Liver (freehand)	3.33 ± 0.99	3.46 ± 0.99	0.94 (0.86–0.97)	3.21 ± 0.74	3.17 ± 0.73	0.99 (0.98–1.00)	3.45 ± 0.77	3.37 ± 0.69	0.96 (0.92–0.98)
Spleen:Liver (volume)	3.65 ± 0.76	3.68 ± 0.74	0.84 (0.67–0.93)	3.72 ± 0.69	3.72 ± 0.77	0.93 (0.84–0.97)	3.73 ± 0.81	3.70 ± 0.74	0.96 (0.90–0.98)
Bone marrow:Liver (freehand)	0.55 ± 0.14	0.55 ± 0.13	0.86 (0.70–0.93)	0.55 ± 0.13	0.54 ± 0.13	0.97 (0.94–0.99)	0.58 ± 0.12	0.56 ± 0.13	0.90 (0.79–0.96)
Bone marrow:Liver (volume)	0.65 ± 0.15	0.65 ± 0.13	0.77 (0.55–0.89)	0.68 ± 0.15	0.68 ± 0.16	0.98 (0.95–0.99)	0.67 ± 0.14	0.64 ± 0.15	0.90 (0.77–0.95)

Malignant lesion and healthy tissue reference measurements (SUV_max_; mean ± SD) and their ratios, of all three raters from two timepoints, with intraclass correlation coefficient (ICC) and its 95% confidence interval (CI). ICC > 0.9 indicates excellent, 0.75 to < 0.9 good, 0.5 to < 0.75 moderate, and < 0.5 poor agreement

*AA* aortic arch, *BP* blood pool, *C* carina, *CI* confidence interval, *ICC* intraclass correlation coefficient, *GOJ* gastroesophageal junction, *L* lung, *LN* lymph node metastasis, *ROI* region of interest, *T* primary lung tumour

Scoring of healthy reference tissue blood pool (*n* = 25) demonstrated good or excellent intra-rater agreement for raters A, B and C (ICCs 0.90, 0.98, 0.97 respectively). The intra-rater ICC for freehand SUV_max_ scores of healthy lung tissue (*n* = 25; ICCs 0.90, 0.86, 1.00) were good or excellent. Applying a 3-cm-diameter sphere volumetric rule-based approach at the level of the aortic arch (ICCs 0.96, 0.96, 0.99) or the carina (ICCs 0.98, 0.94, 0.98) demonstrated excellent intra-rater agreement for all raters. Calculated T:L ratios using freehand and volumetric spheres at the aortic arch or the carina all demonstrated either good or excellent intra-rater agreement for all three raters (Table 3)*.*

There was excellent intra-rater agreement of manual freehand SUV_max_ scores of normal liver (*n* = 25; ICCs 0.94, 0.99, 0.99), spleen (*n* = 25; ICCs 0.91, 0.99, 0.97) and bone marrow (*n* = 25; ICCs 0.91, 0.99, 0.90) for all raters. Applying the rule-based volumetric approach to measuring the SUV_max_ of the liver (ICCs 0.90, 0.98, 0.96), spleen (ICCs 0.95, 0.96, 0.97) and bone marrow (ICCs 0.91, 0.98, 0.91) demonstrated good or excellent agreement. Spleen-to-liver ratio (SLR) measured using freehand (ICCs 0.94, 0.96, 0.99) and volumetric (ICCs 0.84, 0.93, 0.96) approaches both demonstrated good or excellent intra-rater agreement of SUV_max_ scores for raters A, B and C. Similarly, bone marrow-to-liver ratios measured using freehand (ICCs 0.86, 0.97, 0.90) and volumetric (ICCs 0.77, 0.98, 0.90) approaches demonstrated good or excellent intra-rater agreement for all raters.

**Fig. 3:**
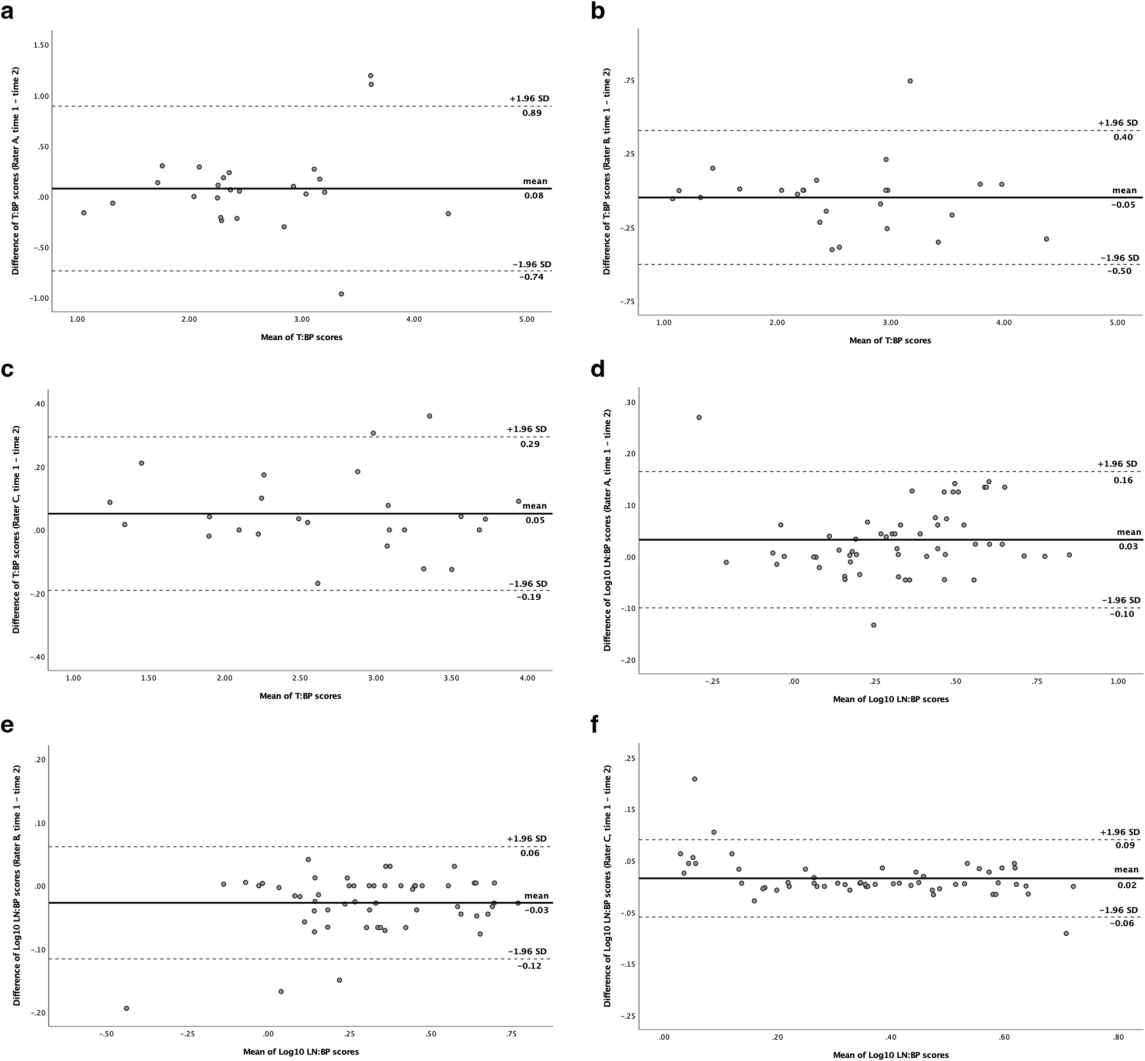
Intra-rater Bland–Altman level of agreement plots for T:BP (**a**–**c**) and log_10_ LN:BP (**d**–**f**) scores. Solid horizontal lines represent between-timepoints mean difference. Upper and lower 95% limits of agreement represented by dashed lines. **a** T:BP scores rater A, time 1 versus time 2 (t-test *p* = 0.35; *β* = 0.11, *p* = 0.36); **b** T:BP scores rater B, time 1 versus time 2 (*p* = 0.3; *β* = -0.03, *p* = 0.64); **c** T:BP scores rater C, time 1 versus time 2 (*p* < 0.05; *β* = -0.01, *p* = 0.71); **d** LN:BP scores rater A, time 1 versus time 2 (*p* < 0.05; *β* = 0.04, *p* = 0.35); **e** LN:BP scores rater B, time 1 versus time 2 (*p* < 0.05; *β* = 0.04, *p* = 0.07); **f** LN:BP scores rater C, time 1 versus time 2 (*p* < 0.05; *β* = -0.09, *p* < 0.05)

Good or excellent intra-rater agreement was demonstrated for T:BP measurements for rater A (ICC 0.86; 95% CI 0.71–0.94), rater B (ICC 0.97; 95% CI 0.92–0.98), and rater C (ICC 0.99; 95% CI 0.97–0.99). LN:BP measurements demonstrated excellent intra-rater agreement for all three raters (ICCs 0.94, 0.98, 0.98). Thoracic metastasis-to-blood pool (ThMet:BP; ICCs 0.95, 0.95, 0.99) and distant metastasis-to-blood pool (DisMet:BP; ICCs 0.89, 0.96, 0.98) ratios also demonstrated good or excellent intra-rater agreement for each rater. Bland–Altman plot analysis demonstrated intra-rater agreement with no proportional bias for T:BP scores for raters A (*β* coefficient = 0.11, *p* = 0.36) and B (*β* = − 0.03, *p* = 0.64) (Fig. 3). However, one-sample *t* test of T:BP scores demonstrated statistical difference between the means of rater C time-point 1 and time-point 2 (*p* < 0.05). Comparison of the means of the separate time-point LN:BP scores were statistically significant for each rater (*p*-values < 0.05). Bland–Altman analysis for ThMet:BP scores for raters A (*β* = 0.2, *p* < 0.05) and C (*β* = 0.11, *p* < 0.05) demonstrated intra-rater agreement but with proportional bias (Additional file 3: Fig. S2). A t-test comparison for rater B demonstrated significantly different ThMet:BP scores between time-point 1 and 2 (*p* < 0.05). Bland–Altman analysis demonstrated intra-rater agreement for all three raters for DisMet:BP scores; however, there was proportional bias on linear regression for rater B (*β* = − 0.15, *p* < 0.05).

## Discussion

In this study, we demonstrate that quantitative assessment of [^99m^Tc]NM-01 SPECT/CT is reliable and reproducible within and between independent raters of variable experience in nuclear medicine. Inter-rater agreement was demonstrated for all malignant lesions (primary lung, lymph node, thoracic and distant metastases) using xSPECT BroadQuantification assessed SUV_max_ scores or their equivalent malignant lesion-to-blood pool ratios of [^99m^Tc]NM-01 SPECT/CT. Additionally, intra-rater agreement was also demonstrated for malignant lesion SUV_max_ scores and their ratios. Measurements of normal tissue using freehand or volumetric approaches demonstrated good or excellent inter- and intra-rater agreement, suggesting simplified and standardised manual measurement techniques (volumetric) could be utilised without negatively impacting the reproducibility of results between and within individual raters and over time. [^99m^Tc]NM-01 uptake measured by T:BP on SPECT/CT has already been shown to correlate with PD-L1 expression measured by immunohistochemistry (*r* = 0.68, *p* = 0.014) [11]. Importantly, that study also documented both inter- and intra-tumoural heterogeneity of uptake. This study’s findings provide further evidence that [^99m^Tc]NM-01 SPECT/CT has the potential for reliably quantifying and as such, clinical utility as a diagnostic agent for PD-L1 assessment. PD-L1 expression is both dynamic and heterogenous, therefore, non-invasive assessment is an attractive possibility, with the potential to better stratify patients for anti-PD-1/PD-L1 therapies, as well as to determine changes of expression in response to therapy and heterogeneity of expression in responding versus non-responding lesions.

Several other radiopharmaceuticals are under development for imaging the PD-1/PD-L1 axis as potential predictive and/or prognostic biomarkers [15]. Uptake of ^18^Fluorine-labelled anti-PD-L1 Adnectin (^18^F-BMS-986192) measured using SUV_peak_ on positron emission tomography (PET)/CT correlated with PD-L1 expression ≥ 50% by immunohistochemistry in NSCLC [16, 17]. Drug-labelled ^89^Zirconium-Atezolizumab, including participants with NSCLC, bladder and breast cancers, demonstrated better prediction of clinical response using mean SUV_max_ on PET compared with either SP263 or SP142 PD-L1 immunohistochemical assays [18]. In both studies, along with several other novel radiopharmaceuticals in early phase clinical trials, both inter- and intra-tumoural heterogeneity of uptake (i.e. PD-L1 expression) was demonstrated (consistent with findings reported in the early phase study of [^99m^Tc]NM-01 SPECT/CT) [11].

In the majority of studies, PET/CT imaging approaches have been taken, with benefits including greater spatial resolution and standardised quantitative assessment. Drug-labelled radiopharmaceuticals offer additional information about drug distribution and are site-binding with the potential of theranostic applications. However, optimal imaging time is usually in the region of days post-administration due to the large size of monoclonal antibodies and longitudinal assessment may be hampered by therapeutic drug binding site occupancy. [^99m^Tc]NM-01 is a small (14.3 kDa) antigen-binding fragment radiopharmaceutical with rapid blood clearance, and optimal SPECT/CT imaging at just 2 h following administration [11, 19]. Pre-clinical studies have demonstrated that it does not directly block the PD-L1 binding site nor interfere with the PD-1/PD-L1 axis, and therefore, it has the potential to assess whole-body PD-L1 status before, during and after anti-PD-1/PD-L1 therapy [19]. The ongoing PECan (NCT04436406) study involves [^99m^Tc]NM-01 SPECT/CT at both baseline and following anti-PD-1/PD-L1 therapy, and aims to demonstrate this in vivo. Whilst tumour-to-background ratios appear to be relatively lower than published examples using PET monoclonal antibody tracers, likely due to a partial sink effect, increasing the nanobody dose from 100 to 400 µg was not associated with any significant differences in tumour to background in the phase 1 study [11]. Despite this, the inter- and intra-rater agreement remains good to excellent.

There are additional benefits to SPECT/CT imaging with both [^99m^Tc] radioisotope and SPECT being widely available and relatively inexpensive. Here we have demonstrated that a quantifiable SPECT/CT approach produces reproducible and reliable [^99m^Tc]NM-01 uptake measurements in malignant NSCLC lesions, as well as in healthy reference tissues. Simple rule-based approaches to measuring healthy reference tissues also demonstrated acceptable inter- and intra-rater agreement, suggesting these methods may be used clinically to standardise and simplify image analysis without any negative impact on quantification.

This study has some limitations, including its sample size; nevertheless, the relatively narrow confidence intervals suggest a reasonable estimate of the agreement. The number of measurable extra-nodal thoracic (lung or pleural) metastases was limited (*n* = 9, all time points) and, as such, the inter- and intra-rater ICCs, although excellent, should be interpreted with more caution. Semi-quantitative SPECT using CT attenuation correction is a relatively novel methodology compared to PET/CT and is likely to be less accurate and more subject to partial volume effects. Nevertheless, with validation, quantifiable SPECT/CT offers the potential for comparison of [^99m^Tc]NM-01 SPECT/CT PD-L1 assessment with alternative PD-L1 PET radiopharmaceuticals.

## Conclusion

Overall, good or excellent inter- and intra-rater agreement of the quantitative assessment of [^99m^Tc]NM-01 SPECT/CT in NSCLC primary and metastatic lesions was demonstrated in this study. As such, there is potential for quantifiable [^99m^Tc]NM-01 SPECT/CT assessment of PD-L1 expression and its inter- and intra-tumoural heterogeneity. With further understanding of the relationship between PD-L1 expression by immunohistochemistry and by [^99m^Tc]NM-01 SPECT/CT, it may also be possible for both quantitative (as described in this study) and qualitative assessments to be made by raters blind to immunohistochemical PD-L1 expression, and their agreement evaluated. Ongoing and subsequent clinical trials are warranted to confirm the utility of [^99m^Tc]NM-01 SPECT/CT in clinical practice.

## Supplementary Information

**Additional file 1: Table S1.** Participant demographics.

**Additional file 2: Fig. S1.** Inter-rater Bland–Altman level of agreement plots for ThMet:BPand log_10_ DisMet:BPscores. Solid horizontal lines represent between-timepoints mean difference. Upper and lower 95% limits of agreement represented by dashed lines. **a** ThMet:BP scores of rater A versus B; **b** ThMet:BP scores of rater A versus C; **c** ThMet:BP scores of rater B versus C; **d** DisMet:BP scores of rater A versus B; **e** DisMet:BP scores of rater A versus C; **f** DisMet:BP scores of rater B versus C.

**Additional file 3: Fig. S2.** Intra-rater Bland–Altman level of agreement plots for ThMet:BPand log_10_ DisMet:BPscores. Solid horizontal lines represent between-timepoints mean difference. Upper and lower 95% limits of agreement represented by dashed lines. **a** ThMet:BP scores rater A, time 1 versus time 2; **b** ThMet:BP scores rater B, time 1 versus time 2; **c** ThMet:BP scores rater C, time 1 versus time 2; **d** DisMet:BP scores rater A, time 1 versus time 2; **e** DisMet:BP scores rater B, time 1 versus time 2; **f** DisMet:BP scores rater C, time 1 versus time 2.

## Data Availability

The datasets used and analysed during the current study are available from the corresponding author on reasonable request. Reuse is permitted where aggregate data will advance research in this field, for example, inter-nation or cross-specialty studies of this kind.

## Abbreviations

BP: Blood pool
BLR: Bone marrow-to-liver ratio
CI: Confidence interval
CT: Computed tomography
DisMet: Distant metastasis
DisMet:BP: Distant metastasis-to-blood pool ratio
ECOG: Eastern Cooperative Oncology Group
GOJ: Gastroesophageal junction
ICC: Intraclass correlation coefficient
IHC: Immunohistochemistry
kDa: Kilodalton
L: Lung
LN: Lymph node
LN:BP: Lymph node-to-blood pool ratio
NM-01: A single-domain antibody targeting PD-L1
NSCLC: Non-small cell lung cancer
PD-1: Programmed cell death protein 1
PD-L1: Programmed death-ligand 1
PET: Positron emission tomography
ROI_max_: Maximum region of interest
SLR: Spleen-to-liver ratio
SPECT: Single-photon emission compute tomography
SUV_max_: Maximum standardised uptake value
SUV_mean_: Mean standardised uptake value
T: Tumour
T:BP: Primary tumour-to-blood pool ratio
[^99m^Tc]: Technetium-99m
ThMet: Thoracic metastasis
ThMet:BP: Thoracic metastasis-to-blood pool ratio
TPS: Tumour proportion score
