# Supplementary material for: Inter-rater and intra-rater agreement of [99mTc]-labelled NM-01, a single-domain programmed death-ligand 1 (PD-L1) antibody, using quantitative SPECT/CT in non-small cell lung cancer
Source: EJNMMI Res. 2023 May 31;13:51. doi: 10.1186/s13550-023-01002-4 (PMC10232393; doi:10.1186/s13550-023-01002-4)
Supplement: Supplementary file 1 — Additional file 1: Table S1. Participant demographics. [file 13550_2023_1002_MOESM1_ESM.docx]

**Supplementary Table 1. Participant demographics.**

| ID | Age (yrs) | Sex | ECOG score | NSCLC tumour histology | TNM staging at diagnosis | Primary tumour size max (mm) | Measured  disease site(s) | SP263 PD-L1 TPS (%) | Administered radioactivity  baseline (MBq/kg) | Administered radioactivity  follow-up (MBq/kg) |
| --- | --- | --- | --- | --- | --- | --- | --- | --- | --- | --- |
| 1 | 63 | M | 1 | Adenocarcinoma | T2aN2M1a | 33 | RLL, thoracic LNs,  Rt pleura | <1 | 7.38 | 6.95 |
| 2 | 59 | M | 1 | Adenocarcinoma | T4N3M0 | 49 | RLL, thoracic LNs | 70 | 7.81 | 11.50 |
| 3 | 53 | F | 0 | Adenocarcinoma | T4N3M1c | 17 | LUL, thoracic LNs,  Lt adrenal, sternum | 55 | 7.93 | -- |
| 4 | 66 | F | 0 | Adenocarcinoma | T1bN0M1b | 27 | RLL, Rt adrenal,  precaval node, Lt bowel | <1 | 11.98 | 13.85 |
| 5 | 58 | F | 0 | Adenocarcinoma | T3NXM1b | 60 | RUL, thoracic LNs, pericardial, ES muscle | 90 | 9.15 | 10.25 |
| 6 | 64 | M | 1 | NOS | T4N3M1a | 47 | Rt central lung, Lt SCF and thoracic LNs | 80 | 7.51 | 8.36 |
| 7 | 73 | M | 1 | Squamous cell carcinoma | T4N2M1x | 64 | Rt lung, satellite lung, thoracic and retrocaval LNs | 55 | 7.45 | 7.76 |
| 8 | 72 | M | 1 | Adenocarcinoma | T1bN2M1b | 29 | Lt lingula, thoracic LNs,  Rt adrenal | 95 | 9.71 | 10.71 |
| 9 | 75 | F | 1 | Adenocarcinoma | T2aN2M1b | 30 | RML, thoracic LNs,  Rt adrenal, L1 bone | <1 | 5.66 | 7.06 |
| 10 | 59 | M | 1 | Adenocarcinoma | T2aN2M1a | 38 | LLL, thoracic LNs | <1 | 9.27 | 6.74 |
| 11 | 63 | M | 1 | NOS | T1cN3M1c | 13 | Lt hilar, thoracic LNs,  Lt adrenal, T5 bone | 100 | 5.51 | 6.14 |
| 12 | 52 | F | 1 | Adenocarcinoma | T1N2M1a | 24 | LUL, thoracic LNs | 0 | 4.66 | -- |
| 13 | 70 | M | 1 | Squamous cell carcinoma | T4N2M1a | 30 | LUL, RUL,  pleural, Lt SCF | 2 | 9.21 | 11.27 |
| 14 | 66 | F | 1 | Adenocarcinoma | T4N3M1c | 40 | RLL, thoracic  and axillary nodes | 90 | 6.09 | -- |

ECOG - Eastern Cooperative Oncology Group Performance score; ES – erector spinae (muscle); L1 – 1^st^ lumbar vertebra; LLL – left lower lobe; LNs – lymph nodes; Lt – Left; LUL – left upper lobe; NOS – not otherwise specified; NSCLC – non-small cell lung cancer; RLL – right lower lobe; RML – right middle lobe; Rt – Right; RUL – right upper lobe; SCF – supraclavicular fossa; T5 – 5^th^ thoracic vertebra; TPS – tumour proportion score; -- denotes second timepoint scan not performed. TNM staging according to 8^th^ Edition of TNM in Lung Cancer. Primary tumour size = maximum length as per RECIST CT measurement.
