# Supplementary material for: Inter-rater and intra-rater agreement of [99mTc]-labelled NM-01, a single-domain programmed death-ligand 1 (PD-L1) antibody, using quantitative SPECT/CT in non-small cell lung cancer
Source: EJNMMI Res. 2023 May 31;13:51. doi: 10.1186/s13550-023-01002-4 (PMC10232393; doi:10.1186/s13550-023-01002-4)
Supplement: Supplementary file 2 — Additional file 2: Fig. S1. Inter-rater Bland–Altman level of agreement plots for ThMet:BPand log10 DisMet:BPscores. Solid horizontal lines represent between-timepoints mean difference. Upper and lower 95% limits of agreement represented by dashed lines. a ThMet:BP scores of rater A versus B; b ThMet:BP scores of rater A versus C; c ThMet:BP scores of rater B versus C; d DisMet:BP scores of rater A versus B; e DisMet:BP scores of rater A versus C; f DisMet:BP scores of rater B versus C. [file 13550_2023_1002_MOESM2_ESM.pdf]

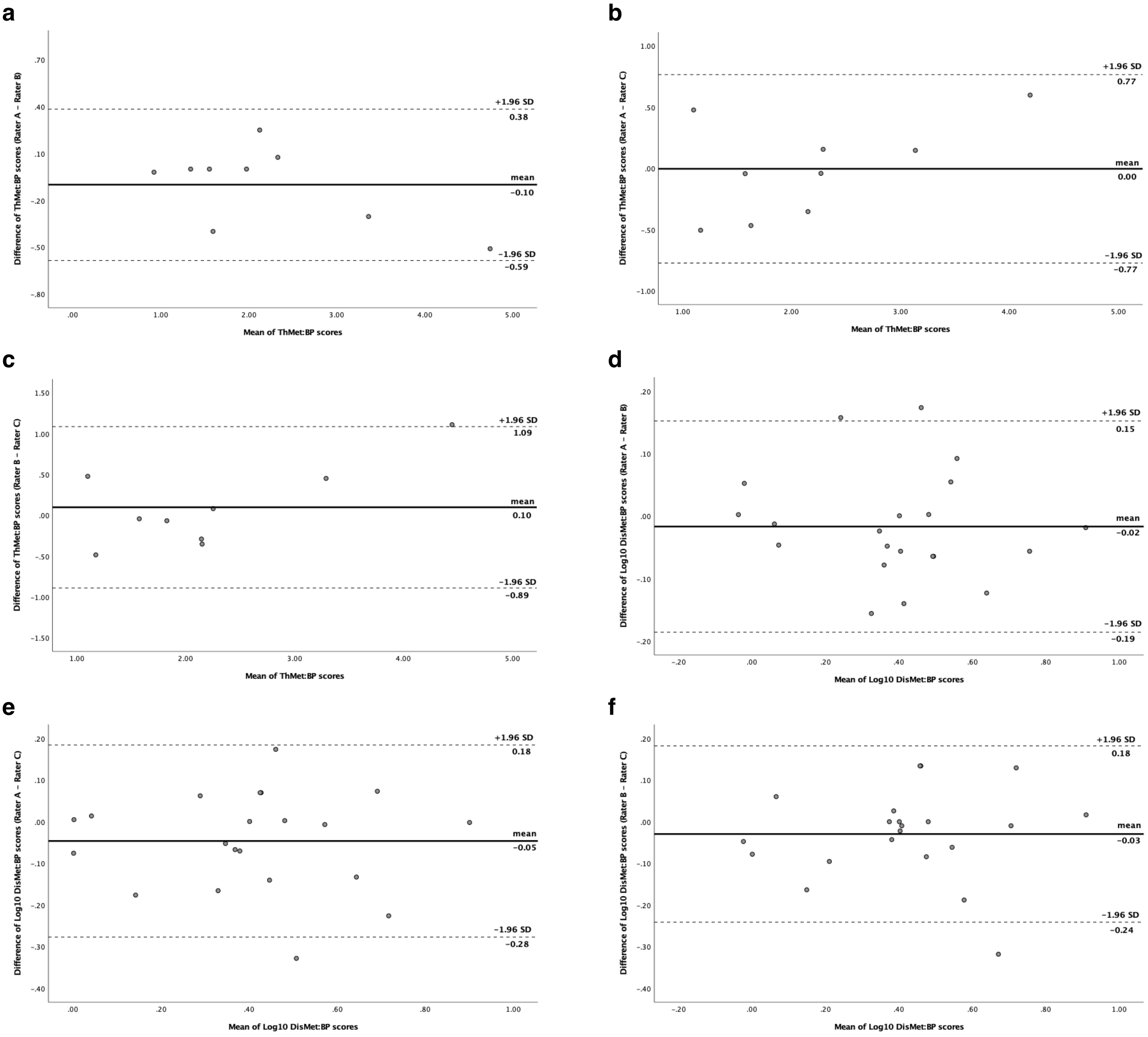

Supplementary Figure 1. Inter-rater Bland-Altman level of agreement plots for ThMet:BP (a-c) and $\log_{10}$ DisMet:BP (d-f) scores. Solid horizontal lines represent between-timepoints mean difference. Upper and lower 95% limits of agreement represented by dashed lines. (a) ThMet:BP scores of rater A vs B (t-test $p = 0.26$; $\beta = -0.13$, $p = 0.09$); (b) ThMet:BP scores of rater A vs C ($p = 0.98$; $\beta = 0.21$, $p = 0.14$); (c) ThMet:BP scores of rater B vs C ($p = 0.58$; $\beta = 0.34$, $p<0.05$); (d) DisMet:BP scores of rater A vs B ($p = 0.38$; $\beta = -0.05$, $p = 0.58$); (e) DisMet:BP scores of rater A vs C ($p= 0.09$; $\beta = -0.02$, $p = 0.85$); (f) DisMet:BP scores of rater B vs C ($p = 0.22$; $\beta = 0.03$, $p = 0.81$).
