# Supplementary material for: Inter-rater and intra-rater agreement of [99mTc]-labelled NM-01, a single-domain programmed death-ligand 1 (PD-L1) antibody, using quantitative SPECT/CT in non-small cell lung cancer
Source: EJNMMI Res. 2023 May 31;13:51. doi: 10.1186/s13550-023-01002-4 (PMC10232393; doi:10.1186/s13550-023-01002-4)
Supplement: Supplementary file 3 — Additional file 3: Fig. S2. Intra-rater Bland–Altman level of agreement plots for ThMet:BPand log10 DisMet:BPscores. Solid horizontal lines represent between-timepoints mean difference. Upper and lower 95% limits of agreement represented by dashed lines. a ThMet:BP scores rater A, time 1 versus time 2; b ThMet:BP scores rater B, time 1 versus time 2; c ThMet:BP scores rater C, time 1 versus time 2; d DisMet:BP scores rater A, time 1 versus time 2; e DisMet:BP scores rater B, time 1 versus time 2; f DisMet:BP scores rater C, time 1 versus time 2. [file 13550_2023_1002_MOESM3_ESM.pdf]

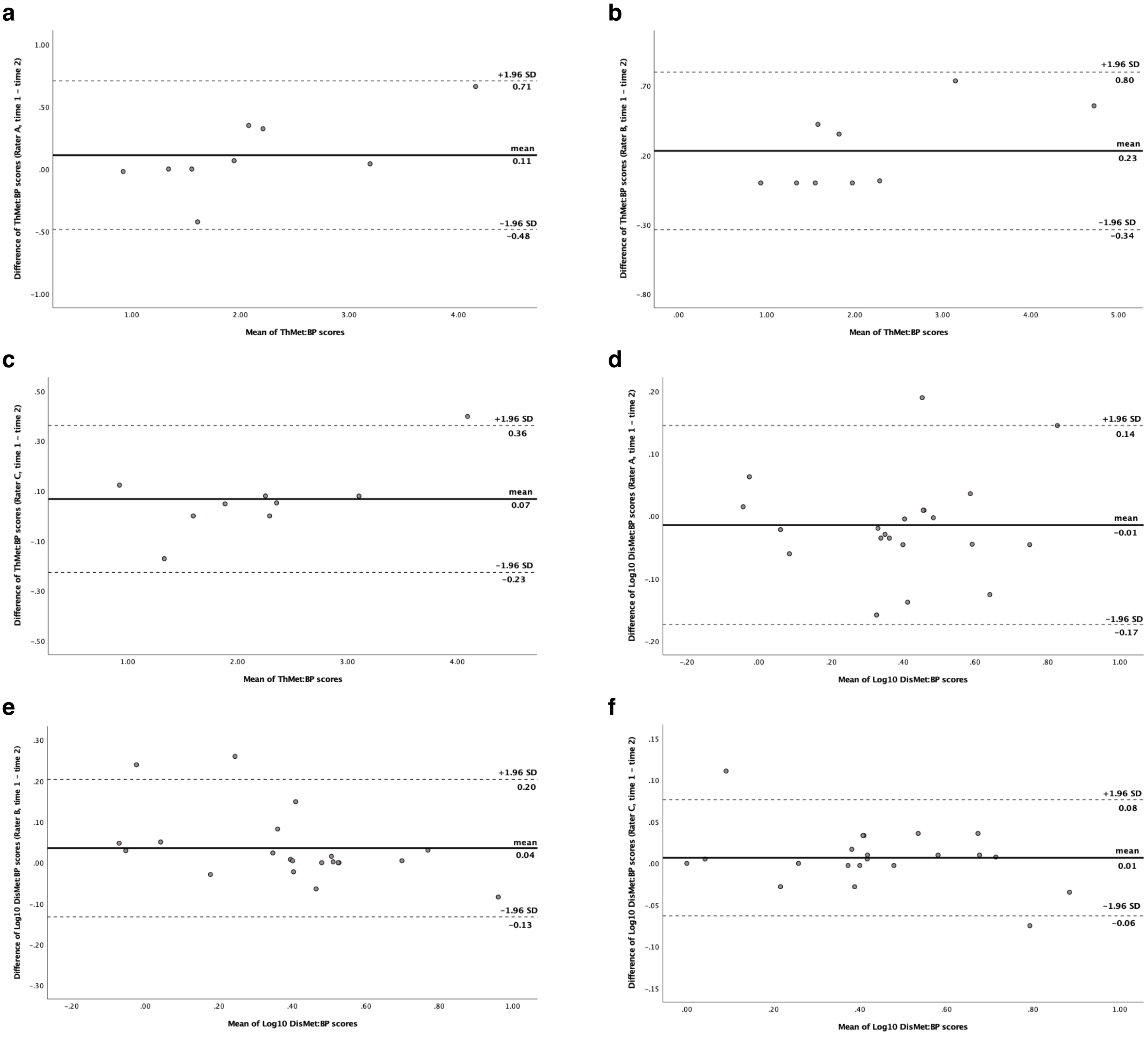

Supplementary Figure 2. Intra-rater Bland-Altman level of agreement plots for ThMet:BP (a-c) and $\log_{10}$ DisMet:BP (d-f) scores. Solid horizontal lines represent between-timepoints mean difference. Upper and lower 95% limits of agreement represented by dashed lines. (a) ThMet:BP scores rater A, time 1 vs time 2 (t-test $p = 0.31$; $\beta = 0.21$, $p<0.05$); (b) ThMet:BP scores rater B, time 1 vs time 2 ($p<0.05$; $\beta = 0.17$, $p<0.05$); (c) ThMet:BP scores rater C, time 1 vs time 2 ($p = 0.21$; $\beta = 0.11$, $p<0.05$); (d) DisMet:BP scores rater A, time 1 vs time 2 ($p = 0.43$; $\beta = 0.03$, $p = 0.72$); (e) DisMet:BP scores rater B, time 1 vs time 2 ($p= 0.07$; $\beta = -0.15$, $p<0.05$); (f) DisMet:BP scores rater C, time 1 vs time 2 ($p = 0.40$; $\beta = -0.05$, $p = 0.11$).
